# Public accountability practices of district health management teams: a realist inquiry in two local health systems in Ghana

**DOI:** 10.1186/1472-6963-14-S2-P134

**Published:** 2014-07-07

**Authors:** Sara Van Belle, Susannah H Mayhew

**Affiliations:** 1 Faculty of Public Health and Policy, London School of Hygiene and Tropical Medicine, London, UK; 2 Department of Public Health, Institute of Tropical Medicine, Antwerp, Belgium

## Background

In today’s local health systems in low- and middle-income countries, district health management teams, together with local authorities, bear the responsibility for health sector performance. This infers not only a responsibility for coordination of the multiple actors in pluralistic health systems and the organisation of services and programmes, but also for ensuring accountability towards the population. This explorative study appraised actual public accountability practices of district health management teams in two local health systems in Ghana and explored how these could be improved.

## Materials and methods

We used a comparative case study design based on realist inquiry principles. An initial middle range theory was developed on the basis of a literature review of governance and accountability spanning different social science research traditions. It was tested in one urban and one rural health system. Data collection included in-depth interviews, informal discussions, and review of documents, reports and routine data.

## Results

In both districts, the health management teams had strong upward accountability systems through which they accounted to the regional and central level authorities of the Ghana Health Service. This accountability was enforced by command-and-control mechanisms such as centrally set priorities and planning directives, audits and performance reviews. In the rural local health system, the district health management team had strong horizontal accountability practices towards the District Assembly and INGOs with whom they collaborate in health service delivery programmes. These relations were based on shared interests and reciprocity. However, in both sites, public accountability strategies or processes were found to be virtually absent. Apart from complaint boxes, there were no formal channels of communication nor for participation of the public in local priority-setting and performance assessment. Procedures through which local health actors could be made accountable towards the public were also found to be absent. As a result, none of the district health management teams achieved full public accountability.

## Conclusions

The study identified ways to improve public accountability. There is a critical need for more transparency and information sharing with the public. The existing performance appraisals, for instance, should be opened to representatives of the public. Enforcement of accountability practices can be improved by an agent who takes up the role of meta-governor and holds all actors accountable. This role could be played by the district assembly. Channels for effective remedial action need to be created, including health facility boards in which representatives of the public participate.

